# Longitudinal increases in childhood depression symptoms during the COVID-19 lockdown

**DOI:** 10.1136/archdischild-2020-320372

**Published:** 2020-12-09

**Authors:** Giacomo Bignardi, Edwin S Dalmaijer, Alexander L Anwyl-Irvine, Tess A Smith, Roma Siugzdaite, Stepheni Uh, Duncan E Astle

**Affiliations:** MRC Cognition and Brain Sciences Unit, University of Cambridge, Cambridge, UK

**Keywords:** psychology, adolescent health

## Abstract

**Objective:**

There has been widespread concern that so-called lockdown measures, including social distancing and school closures, could negatively impact children’s mental health. However, there has been little direct evidence of any association due to the paucity of longitudinal studies reporting mental health before and during the lockdown. This present study provides the first longitudinal examination of changes in childhood mental health, a key component of an urgently needed evidence base that can inform policy and practice surrounding the continuing response to the COVID-19 pandemic.

**Methods:**

Mental health assessments on 168 children (aged 7.6–11.6 years) were taken before and during the UK lockdown (April–June 2020). Assessments included self-reports, caregiver reports, and teacher reports. Mean mental health scores before and during the UK lockdown were compared using mixed linear models.

**Results:**

A significant increase in depression symptoms during the UK lockdown was observed, as measured by the Revised Child Anxiety and Depression Scale (RCADS) short form. CIs suggest a medium-to-large effect size. There were no significant changes in the RCADS anxiety subscale and Strengths and Difficulties Questionnaire emotional problems subscale.

**Conclusions:**

During the UK lockdown, children’s depression symptoms have increased substantially, relative to before lockdown. The scale of this effect has direct relevance for the continuation of different elements of lockdown policy, such as complete or partial school closures. This early evidence for the direct impact of lockdown must now be combined with larger scale epidemiological studies that establish which children are most at risk and tracks their future recovery.

## Abstract (precis)

Depressive but not anxiety symptoms increased in children aged 7-11 years in the first UK lockdown (April to June 2020).

**What is already known on this topic?**

- Due to a lack of prospective studies with before-lockdown assessments, the impacts of physical distancing and school closures on children’s mental health is unclear.
- Adolescence is a developmental period where mental health may be particularly vulnerable to reduced peer interaction and loneliness.
- Existing research in adult populations suggests deteriorations in mental health during the lockdown policies in different countries.

**What this study adds?**

- Changes in three mental health scales before and during the lockdown are analysed used mixed linear models in a UK cohort of 8–12 year olds.
- Depression symptoms increased during lockdown, with CIs suggesting a medium-to-large standardised mean difference, even when controlling for age at assessment.
- Changes in anxiety and emotional problems were small and not statistically significant, suggesting that depression may be particularly susceptible.

## Background

In response to the COVID-19 pandemic, the UK Government implemented a national ‘lockdown’ involving school closures and social distancing. There has been widespread concern that these measures will negatively impact child and adolescent mental health.^1–3^ To date, however, there is relatively little direct evidence of this. This is in part due to the paucity of studies including pre-lockdown baseline data. Longitudinal changes within the same individuals represent the most direct way of quantifying the association between the onset of lockdown and children’s mental health.

There is good reason to suspect that the implementation of a lockdown has negatively impacted children’s mental health. Early evidence from adult cohort studies suggests that there have been clinically meaningful deteriorations in anxiety, mental health and well-being during lockdown.^4–7^ One study suggests younger and lower income individuals have been more affected.^8^ However, one longitudinal study from a large Dutch probability sample of adults found a very small, non-significant change in depression and anxiety, measured with the five-item Mental Health Index.^9^

Far less is known about how young children have coped during lockdown, though evidence is rapidly emerging for adolescents. A large, longitudinal study of 13–14 year olds in the UK reported a mixed pattern of changes in well-being, depression and anxiety in April/May 2020 compared with October 2019.^10^ Self-reported well-being and anxiety slightly improved during the lockdown compared with before. However, the analyses do not control for age at assessment or report mean change scores for depression and anxiety for the whole sample.

How the lockdown measures impact children’s mental health may depend on a variety of factors. Loneliness in children is associated with subsequent mental health problems, particularly depression.^11^ Reduced access to play and activities for young people may impair mood homeostasis, engaging in pleasurable activities to improve mood.^12^ There is a particular concern for children already struggling with mental health issues, where access to mental health services has been impacted.^13 14^ Using social media may also mitigate the impacts of physical distancing.^3^ Its also plausible that the alleviation of school-related stressors may elicit short-term improvements, although we are not aware of any existing evidence yet.

We report results from the Resilience in Education and Development (RED) study, a small but rich dataset collected from a cohort of children living in the East of England.^15^ This cohort had been assessed via a combination of caregiver, teacher and child reports of mental health, alongside a variety of other measures. Around 18 months after this initial assessment, these children were subject to the national lockdown. During lockdown, we contacted a subsample of the families and tested for any changes in their children’s mental health and well-being. This study aims to test whether changes in emotional well-being, anxiety and depression occurred during lockdown since the initial assessment.

## Methods

### Participants

The RED study comprises two groups. A larger school group assessed in classrooms (n=567, from 22 classes, 6 schools) and a smaller group of children (N=92) who completed the same and additional assessments at our laboratory. Both samples are convenience samples. Families in the lab group were recruited via posters, word of mouth and online Facebook advertisements. In the school groups, all children in year 3 and 4 classroom groups were recruited into the study using opt-out parental consent. Due to ethical constraints, schools did not provide information on the number of ‘opt-outs’ from their schools. Children absent on the day of before-lockdown testing (ie, due to sickness) will not have data on child-reported mental health. Schools did not provide information on whether children have moved by the time of lockdown.

Baseline assessments occurred between June 2018 and March 2019 in the school group and December 2018 and September 2019 in the laboratory group. Six schools were recruited to take part in the study, with all children in eligible year 3 and 4 classroom groups eligible to take part. Eligibility criteria in the lab group included a medical screener for suitability to undergo medical resonance imaging.

The mental health assessments by caregivers and teachers, both before and during lockdown, were completed using an online survey. Participation was incentivised with a £5 Amazon voucher for completion. We directly contacted all legal caregivers of children in the lab group, and five schools contacted caregivers in the school group, to complete the survey. One school (representing 84 children with baseline data) did not contact caregivers.

One hundred and sixty-eight parents completed mental health assessments for their children during lockdown (142 mothers and 26 fathers, no responders selected ‘grandparent’ or ‘other’), for whom prior mental health data were available. This represents 29% of the contacted, eligible sample. Demographic features of this sample are summarised in table 1.

**Table 1:** Demographic data for both subgroups

	School group	Lab group
Sample size		
N	114	54
Gender		
Male	58	22
Female	56	32
Age at baseline		
Mean	8.7	8.5
SD	0.63	0.66
Age at lockdown assessment		
Mean	10.5	9.4
SD	0.74	0.78
Caregiver is homeowner		
% (N)	73% (83)	63% (34)
Caregiver has degree		
% (N)	64% (67)	60% (32)
Number of responses	105	53
Index of Multiple Deprivation		
Mean decile	7.9	6.9

Only children included in one of the mixed linear model analyses (with both baseline and during lockdown mental health data) are presented here.

### Measures

Three mental health measures were used: the Strengths and Difficulties Questionnaire (SDQ), Emotional Problems subscale and RCADS-short form subscales for depression and anxiety.^16 17^ All scales were adapted for computerised testing using continuous slider scales.

Before lockdown, teachers and caregivers completed the SDQ for the school and lab groups, respectively. At this time point, children in both groups completed the RCADS, along with caregivers in the lab group. Children completed the RCADS on a custom-developed tablet application,^15^ which included audio presentation of each question. Follow-up testing during lockdown took place between 29 April and 19 June 2020, with only caregivers completing the assessments.

We collected several demographic variables from families. Neighbourhood deprivation was estimated using the English Indices of Deprivation, a national statistics database that ranks small areas in England from most (1) to least (10) deprived deciles.^18^ Free school meal eligibility, a widely used proxy for socioeconomic status (SES), measures whether parents are eligible for a series of government benefits.^19^ Caregiver education and homeownership were also assessed in the lockdown questionnaire.

### Statistical analysis

**Figure 1:**
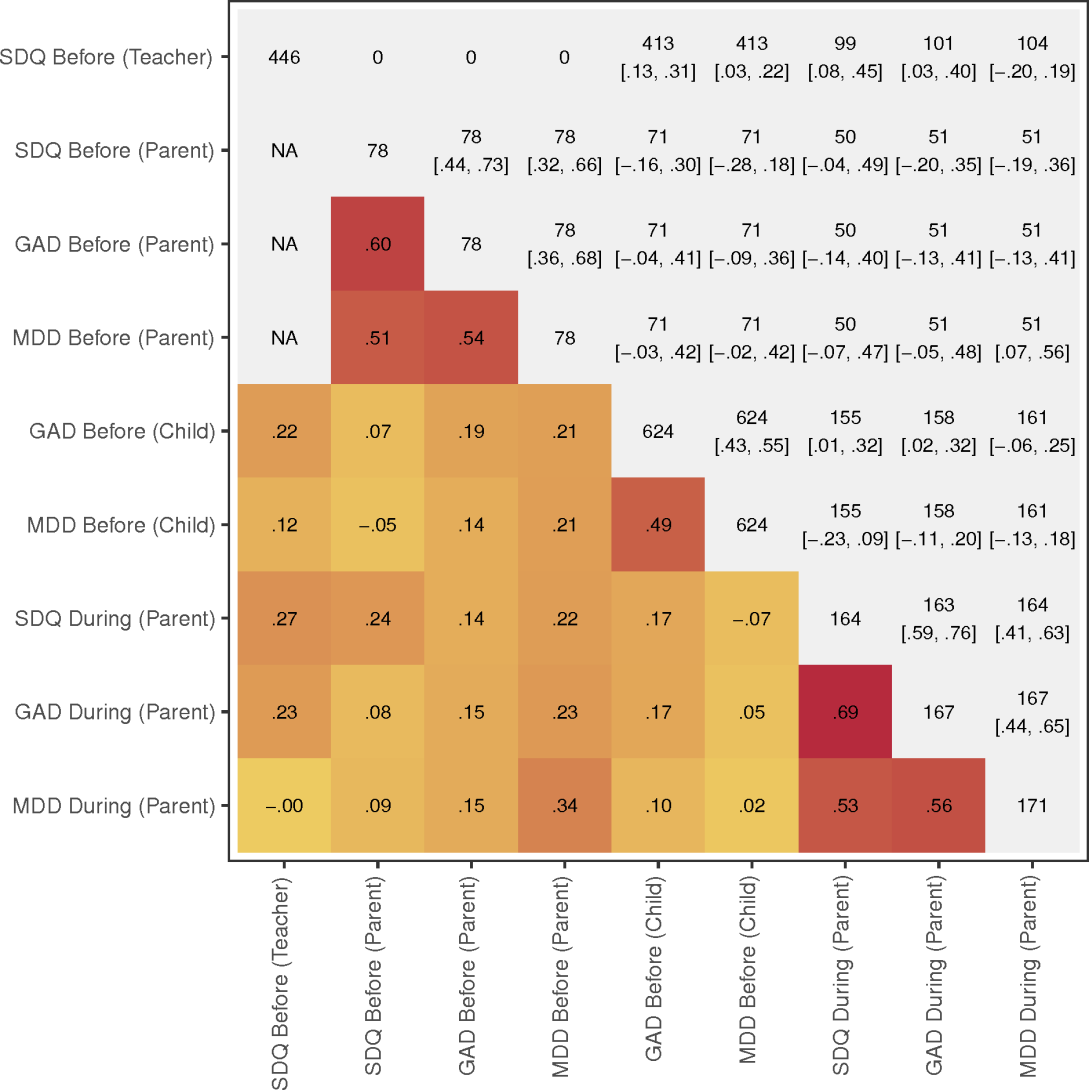
Correlations between mental health variables and patterns of missing data. Below diagonal: Pearson correlations between SDQ Emotional Problems (SDQ), RCADS anxiety subscale (generalised anxiety disorder (GAD)) and RCADS depression subscale (major depressive disorder (MDD)), before and during lockdown. On diagonal: number of observations for each variable. Above diagonal: number of observations with data on two given variables and 95% CIs for correlations. RCADS, Revised Child Anxiety and Depression Scale; SDQ, Strengths and Difficulties Questionnaire.

We report descriptive statistics and correlations between measures in figure 1, which includes all participants. We analysed the impact of lockdown by combining child, teacher and caregiver reports using linear mixed models. Coefficients estimated the effect of lockdown (0=before/1=during lockdown), and responder (0=child/1=caregiver or 0=teacher/1=caregiver), on children’s mental health, including a random intercept for participant. Children were included in a given mixed linear model only if data from before and during lockdown were available for a given mental health outcome. Participant’s age, gender and SES were controlled in sensitivity analyses. SES was measured using a mean of: household income, homeownership, caregiver education and neighbourhood deprivation. SES was scaled to have zero mean and unit variance. Interaction effects between age, gender and SES and lockdown status were examined, by multiplying lockdown status with these variables and entering them into the mixed model.

Analyses were performed using R (V.3.6.2) and the nlme (V.3.1–144) package.^20 21^ Mental health variables were scored using the arithmetic mean response, after recoding each item so that a higher score indicated worse mental health. For mixed linear models, all three mental health outcomes were quantile-normalised to match a standard normal distribution. Normalisation was performed for each outcome after transforming data into a ‘long’ format, with repeated measurements (including before and during lockdown from all raters) gathered in a single variable. Variables were converted into a percentile rank, and we then applied the standard normal distribution quantile function.

### Role of the funding source

The funders had no role in the study design, data collection, data analysis, data interpretation or writing of the manuscript. The corresponding author had full access to all the data and had the final responsibility for the decision to submit for publication.

## Results

First, the baseline data enabled us to estimate the size of any recruitment biases in those who responded during lockdown. There was a small bias for more affluent families to participate. Neighbourhood deprivation (measured using the Index of Multiple Deprivation) and free school meal eligibility weakly predicted non-participation (*r*=−0.17 and −0.18, respectively).^18^ Greater child-reported RCADS depression (r=−0.08, 95% CI −0.16 to 0.01) and anxiety (r=−0.09, 95% CI −0.16 to 0.01) symptoms at baseline also weakly predicted non-participation. The date of lockdown questionnaire completion had weak, non-significant correlations with the three mental health outcomes during lockdown (all |r|<0.06).

Correlations and 95% CIs between all variables are provided in figure 1, alongside patterns of available data and missingness. All three mental health measures during lockdown were strongly correlated (0.53≤*r* ≤ 0.69), though correlations between mental health reports before and during lockdown were generally low (*r*≤0.34). Internal consistency of each mental health scale was above 0.74 for all measures except child-rated depression symptoms (Cronbach’s alpha=0.52).

Table 2 reports all main effects. From the mixed linear models with no control variables, we estimated a non-significant decrease of 0.25 in SDQ emotional problems (B=−0.25, 95% CI −0.54 to 0.05) and a decrease of 0.06 in RCADS anxiety scores (B=−0.06, 95% CI −0.34 to 0.23) during lockdown compared with before. Note that because all outcomes are standardised, the coefficients estimated from mixed linear models (B) can be interpreted similarly to a standardised mean difference like Cohen’s d (see note in table 2).^22^ The CI upper limits suggest that at most a small increase in these symptoms occurred during lockdown. This is consistent with the proportion of children with SDQ emotional problem scores in the elevated range, which changed very little, decreasing from 13% (19 children) to 8% (12 children) from before to during lockdown.^16^ The short-form RCADS subscales do not have established cut-offs for identifying elevated scores.

**Table 2:** Main effects from mixed linear models

	SDQ emotional problems					RCADS anxiety subscale					RCADS depression subscale				
	B	95% CI		P value	N	B	95% CI		P value	N	B	95% CI		P value	N
Model 1															
Lockdown	−0.246	−0.542	0.050	0.103	298/149	−0.055	−0.335	0.225	0.699	371/162	0.736	0.458	1.014	<0.001	377/165
Responder	0.276	−0.056	0.607	0.102	298/149	−0.796	−1.077	−0.515	<0.001	371/162	−1.331	−1.610	−1.052	<0.001	377/165
Model 2															
Lockdown	−0.161	−0.537	0.215	0.398	298/149	0.059	−0.284	0.402	0.736	371/162	0.580	0.239	0.920	0.001	377/165
Responder	0.274	−0.059	0.607	0.106	298/149	−0.796	−1.077	−0.514	<0.001	371/162	−1.302	−1.580	−1.023	<0.001	377/165
Gender	−0.025	−0.351	0.301	0.881	298/149	−0.033	−0.289	0.224	0.802	371/162	−0.328	−0.573	−0.082	0.009	377/165
Lckdwn*Gndr	−0.153	−0.552	0.245	0.449	298/149	−0.223	−0.580	0.134	0.220	371/162	0.249	−0.106	0.604	0.169	377/165
Model 3															
Lockdown	−0.111	−0.505	0.284	0.580	296/148	0.108	−0.238	0.455	0.538	363/158	0.722	0.376	1.068	<0.001	369/161
Responder	0.331	−0.006	0.668	0.055	296/148	−0.790	−1.069	−0.510	<0.001	363/158	−1.320	−1.602	−1.037	<0.001	369/161
Age	−0.013	−0.281	0.256	0.925	296/148	0.003	−0.212	0.218	0.979	363/158	0.027	−0.182	0.236	0.798	369/161
Lockdown*age	−0.215	−0.511	0.081	0.153	296/148	−0.222	−0.477	0.034	0.089	363/158	−0.031	−0.289	0.228	0.816	369/161
Model 4															
Lockdown	−0.255	−0.551	0.041	0.090	298/149	−0.060	−0.340	0.220	0.674	371/162	0.733	0.455	1.011	<0.001	377/165
Responder	0.290	−0.040	0.620	0.085	298/149	−0.792	−1.073	−0.510	<0.001	371/162	−1.329	−1.608	−1.050	<0.001	377/165
SES	−0.140	−0.301	0.020	0.086	298/149	−0.060	−0.187	0.067	0.353	371/162	−0.051	−0.174	0.071	0.409	377/165
Lockdown*SES	−0.046	−0.245	0.153	0.650	298/149	−0.054	−0.233	0.126	0.557	371/162	−0.063	−0.241	0.115	0.487	377/165
Paired t-test															
Lockdown	−0.195	−0.480	0.089	0.173	50	0.145	−0.136	0.426	0.305	51	0.713	0.432	0.994	<0.001	51

N for mixed linear models gives the (number of observations)/(number of individuals). Continuous variables of age and SES were z-scored, and lockdown and responder are binary variables. Lockdown is coded as before (0) or during (1) lockdown. Responder is coded teacher/child (0) or caregiver (1). Gender is coded as male (0) or female (1). Coefficients for binary variables (eg, responder, lockdown and gender) can be interpreted mean group differences. For example, B_gender_=−0.025 indicates that when accounting for lockdown and responder, on average girls scored 0.025 less than boys. Because outcomes are standardised (M=0, SD=1), regression coefficients for binary variables can be interpreted similarly to a standardised mean difference. Sample sizes are lower in model 3 due to missing age information for some children.

RCADS, Revised Child Anxiety and Depression Scale; SDQ, Strengths and Difficulties Questionnaire; SES, socioeconomic status.

**Figure 2:**
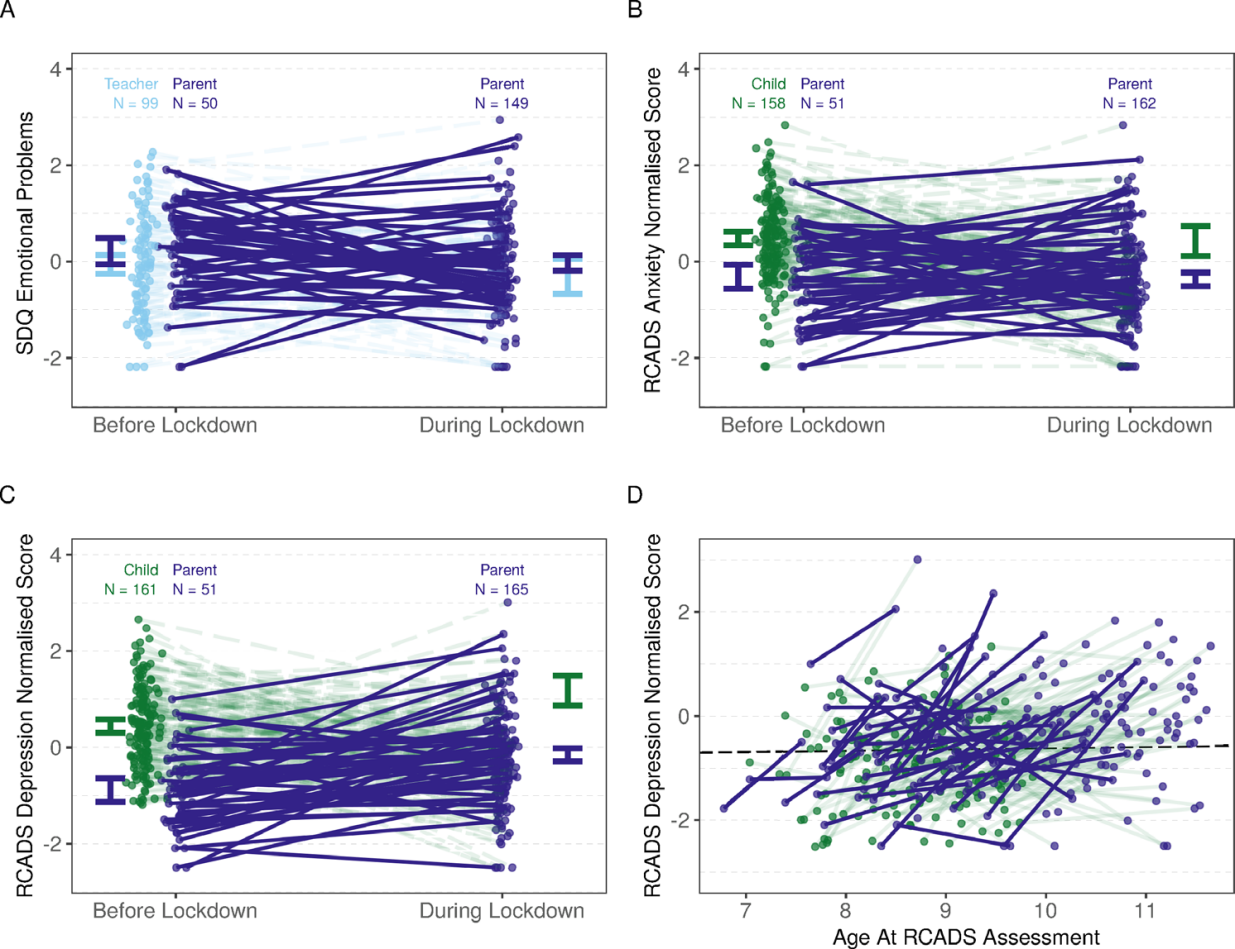
Change in mental health ratings from before to during the lockdown measures. Panels A–C display changes in mental health ratings for all three outcomes, respectively. Dark purple lines indicate changes in only parent-reported mental health scores. Dashed lines indicate changes in mental health scores from either teacher or child reports (before lockdown) to parent report (during lockdown). In each plot, we report the number of responses before and during lockdown, by teachers, children or parents. Panel D plots the same data as panel C, however with age at assessment on the horizontal axis and lines showing individual changes in depression symptoms. This shows a relatively sharp increase in depression symptoms from before to during lockdown, compared with the relatively weak effect of age on depression symptoms (shown in the black, dashed line) estimated from the mixed linear model. In panel D, child-reported mental health measures are reduced by ~1.3 to aid visualisation, as the model estimated that children reported higher depression compared with parents on by this amount on average. RCADS, Revised Child Anxiety and Depression Scale; SDQ, Strengths and Difficulties Questionnaire.

In contrast, standardised RCADS depression scores were on average 0.74 (95% CI 0.46 to 1.01) higher during lockdown than before (see figure 2). The CIs suggest a medium-to-large increase is likely.

Controlling for demographic factors separately (age, gender and SES) did not strongly alter our estimates for these effects. Interaction effects of these three factors were also estimated to assess whether changes in mental health disproportionally occurred in certain groups. No interaction effects were statistically significant, although these estimates are highly uncertain.

A sensitivity analysis using only caregiver-rated mental health before and during lockdown was performed (see bottom table 2). One-sample t-tests were conducted on the standardised change scores, that is, the raw score during lockdown minus the before-lockdown score, divided by the change score SD. These analyses found similar effects as the mixed models (see table 2).

**Figure 3:**
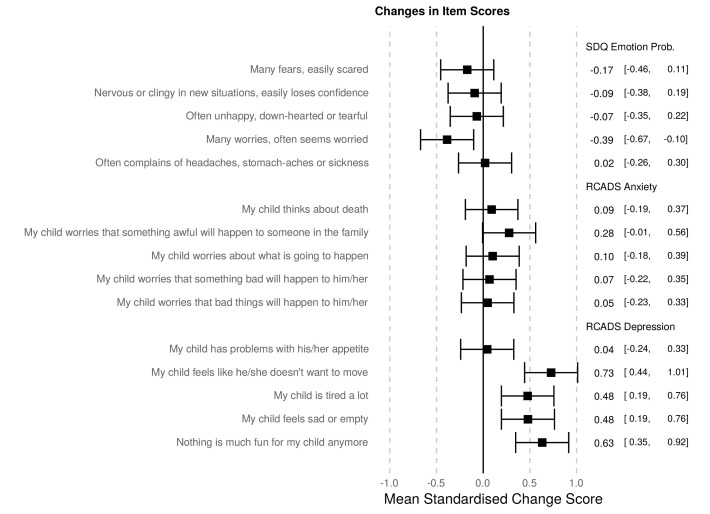
Mean standardised change score and CIs for each mental health question, comparing during to before lockdown, using solely caregiver reported mental health at both time points. Positive numbers indicate worsening of symptom during lockdown. Results support the interpretation that RCADS depression items have increased during lockdown, with more minor changes in other symptoms. RCADS, Revised Child Anxiety and Depression Scale; SDQ, Strengths and Difficulties Questionnaire.

One potential limitation of using mean scale scores is that changes during lockdown may be driven by specific items within the scale. Therefore, changes in responses to each individual question in the mental health scales were examined, using the same t-test approach outlined above (see figure 3). Four out of five of the depression questions showed significant increases during lockdown. Only one other question (‘Many worries, often seems worried’, from the SDQ) significantly changed, decreasing during lockdown (fewer worries during lockdown).

## Discussion

National lockdowns with mass school closures are unprecedented, and the evidence base to guide future policymaking is emerging rapidly. Longitudinal data form a vital component of that evidence base. This study is one of the first longitudinal studies and suggests that children’s depression ratings significantly increased during the lockdown, relative to 18 months beforehand, with a medium-to-large effect. Note that this represents an average and not uniform change across children. The effect of lockdown on mental health did not significantly differ across demographic groups in moderation analyses examining children’s age, gender and family SES. However, larger sample sizes are required to adequately statistically power moderation analyses.^23 24^

### Implications for policy makers and practitioners

The backdrop is that children’s mental health appears to be worsening across successive cohorts, and even before lockdown, the resources for Child and Adolescent Mental Health Services were stretched thin.^25 26^ The current findings suggest that lockdown measures will likely exacerbate this, specifically with an increase in childhood depression symptoms, something previously relatively uncommon in children of this age.^27^ The education sector and families may bear the initial brunt of this.^28^ Indeed, one study has also reported an increase in parent’s psychological symptoms over lockdown.^29^ A key implication of the current findings is that the potential association between lockdown and childhood mental health should be incorporated in the decision-making process of policy makers. When children return to school, their well-being, socialisation and enjoyment are paramount. Additional resources and training will likely be required to equip school teachers in how to support children with low mood and to increase their awareness of referral pathways for professional support.

### Future directions

Future work should follow children over longer time periods to assess long-term effects. First, because there is potential for ‘sleeper effects’ (effects that emerge sometime after an initial adversity, often in a different phase of development), and second, because we need to test whether children’s mood rebounds when school resumes.^30^ Larger cohorts with greater statistical power are needed to address whether the epidemic has had disproportionate effects on particular children and households. Of particular concern are children with existing mental health and other needs. Initial reports have highlighted challenges during school closures facing children with autism and attention deficit hyperactivity disorder.^31 32^

Finally, our analysis of individual questionnaire items suggests that particular symptoms may be differentially affected by the lockdown. Larger epidemiological studies could further explore this potential differential association between lockdown on mental health. One study in 80 Dutch students reported preliminary evidence that global mental health problems did not increase across 2 weeks of the COVID-19 pandemic, but depressive symptoms specifically increased and anxiety symptoms decreased.^33 34^ Studies that only measure mental health using broad, brief mental health measures may fail to detect more specific effects.

### Study limitations

The small sample size of the current study is a limiting factor, which reduces the statistical power and precision of estimates. Therefore, the lack of a statistically significant effect on SDQ scores or RCADS anxiety scale, or moderation effects, should be interpreted with caution. The current study does not have the statistical power to detect small but clinically meaningful changes. Second, because this is a convenience sample collected within our main cohort, the proportion of responders is relatively small compared with the size of the overall cohort. This is perhaps to be expected given the timing of our survey and the context of the pandemic. However, the baseline characteristics were only very weakly associated with which families responded to our invitation to take part. As we only sampled a small region of the UK, caution should be applied in generalising the results to different populations. Third, the mixture of reporters is a limitation for the study, as well as the lack of child-reported measures during lockdown. Children and adults report mental health symptoms differently. This is why reporter is directly incorporated within the model, and the effects are subsequently replicated in a subsample with just longitudinal caregiver report.

## Conclusions

We report longitudinal evidence for the negative association between UK lockdown measures and child mental health. Specifically, we observed a statistically significant increase in ratings of depression, with a medium-to-large effect size. Our findings emphasise the need to incorporate the potential impact of lockdown on child mental health in planning the ongoing response to the global pandemic and the recovery from it.
